# Supplementary material for: ViralMap: predicting features in viral proteins from primary sequence
Source: J Virol. 2026 Jul 28;100(8):e00757-26. doi: 10.1128/jvi.00757-26 (PMC13483385; doi:10.1128/jvi.00757-26)
Supplement: Supplementary file 1 [file jvi.00757-26-s0001.pdf]

SUPPLEMENTAL INFORMATION

ViralMap: Predicting Features in Viral Proteins from Primary Sequence

Shrish Dwivedi, Shaunak Kar, Andrew P. Horton, Jimmy D. Gollihar

Supplementary Figures

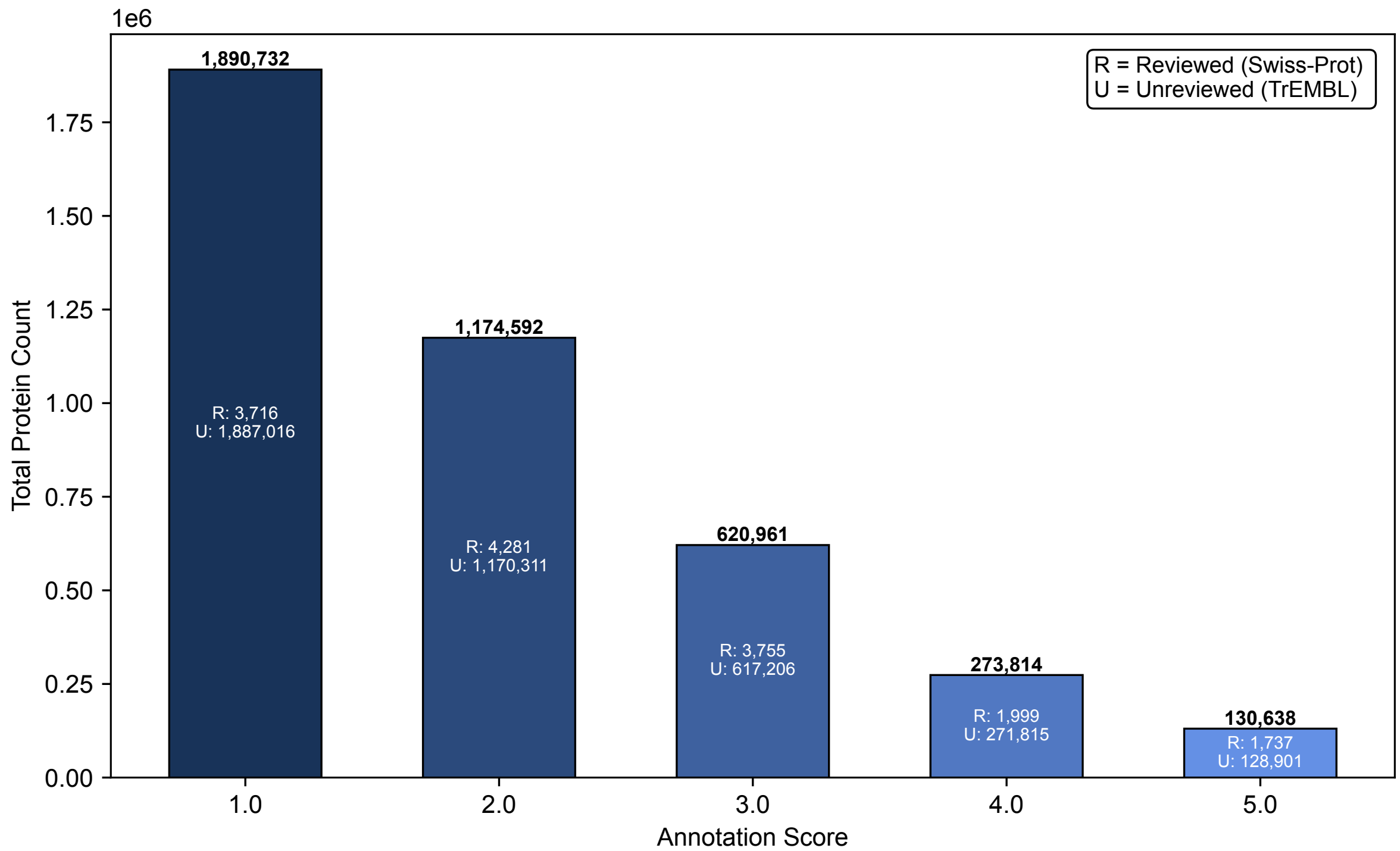

Supplementary Figure 1. The initial download of non-phage viral proteins (n=4,090,737 proteins) from UniProt was composed mainly of poorly annotated entries, with most sequences assigned UniProt annotation scores of 3.0 or lower. The Swiss-Prot (reviewed) designation did not necessarily indicate that the protein was well annotated, since most proteins with a 5.0 annotation score in the dataset were TrEMBL (unreviewed) records.

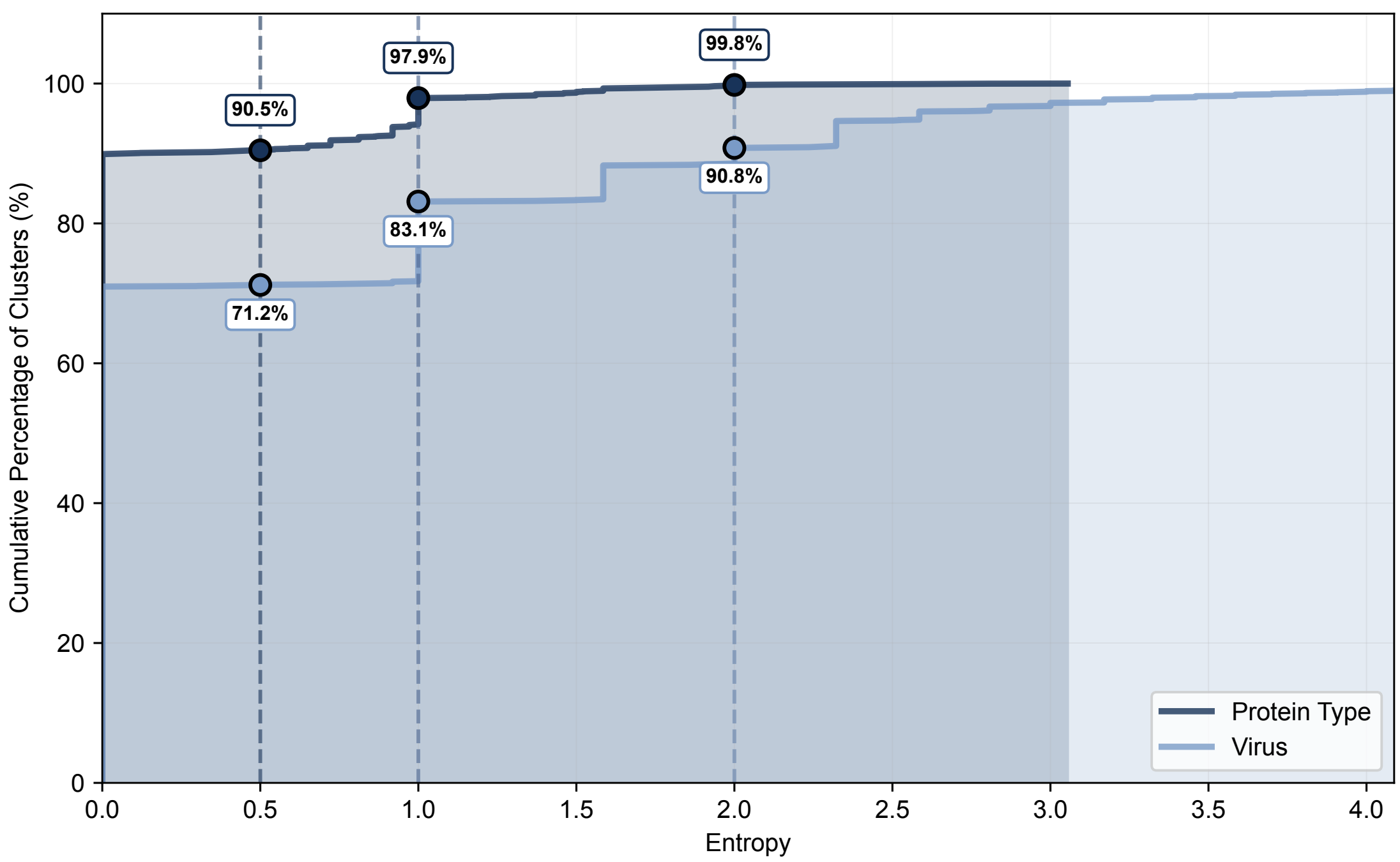

Supplementary Figure 2. MMSeqs2 clustering (60% sequence identity and 50% bidirectional coverage) produces largely homogeneous protein-type (e.g., glycoprotein, matrix protein, etc.) and virus-level downselection clusters. Shown are cumulative distributions of Shannon entropy for UniProt protein names and UniProt organism (virus). The entropy metric shows how similar UniProt entries are within a given cluster with respect to protein type and virus, on a scale from 0 to infinity. For protein type, 99.8% of all clusters have entropy ≤ 2.0, indicating strong homogeneity in protein types. Virus-level homogeneity was similarly high, with 90.8% of clusters having entropy ≤ 2.0. These results suggest that most clusters group variants of the same viral protein, enabling representative selection as described in **Supplementary Note 1**.

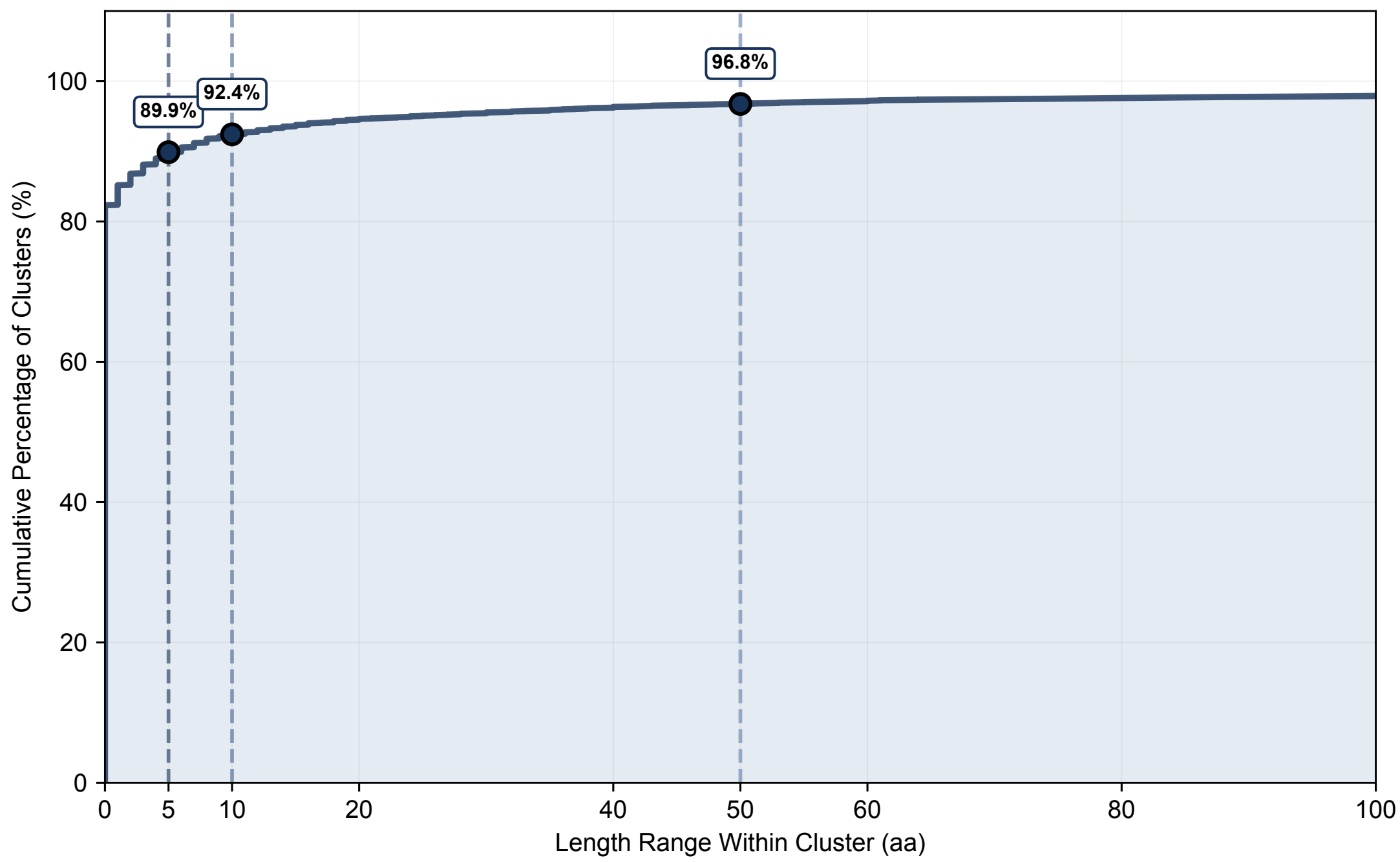

Supplementary Figure 3. Intra-cluster protein length variation is minimal for the majority of MMSeqs2 downselection clusters. Shown is the cumulative distribution of sequence length range (max length – min length) within clusters. Most clusters exhibited narrow length variation, with 89.9% having a length range ≤5 amino acids and 96.8% ≤ 50 amino acids. This indicates that clustered proteins are mostly consistent in length, supporting their use as interchangeable candidates during representative selection **(Supplementary Note 1)**.

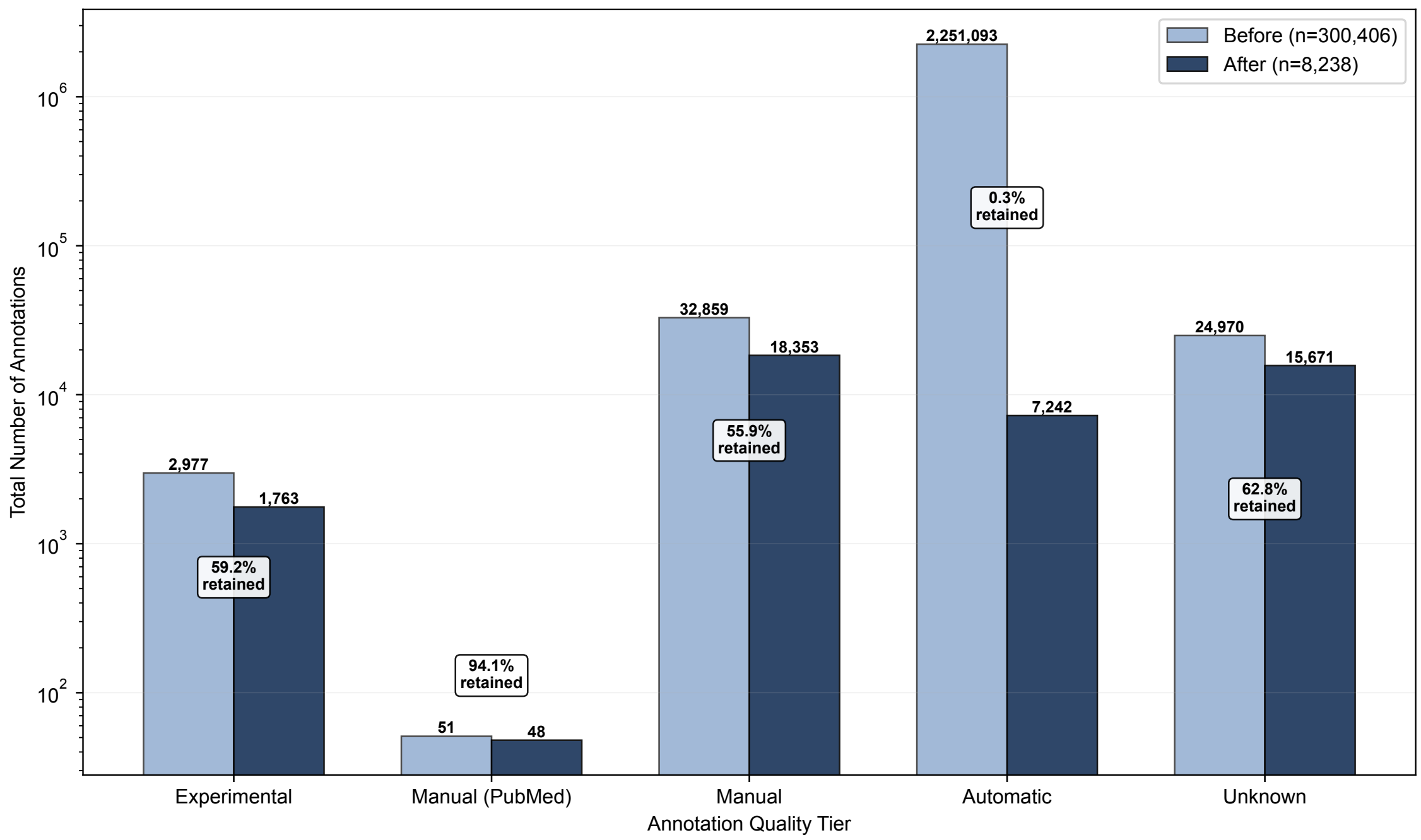

Supplementary Figure 4. Annotation retention by evidence tier following representative selection via Algorithm 1 **(Supplementary Note 1)**. Grouped bars show annotation counts before (n=300,406 proteins) and after (n=8,238 proteins) selection. Retention rates varied by evidence quality: experimental (59.2%), manual with PubMed (94.1%), manual (55.9%), automatic (0.3%), and unknown (62.8%). The algorithm successfully prioritized selecting proteins with higher-quality annotations across all classes while reducing redundancy and preferentially eliminating proteins that contributed automatic annotations.

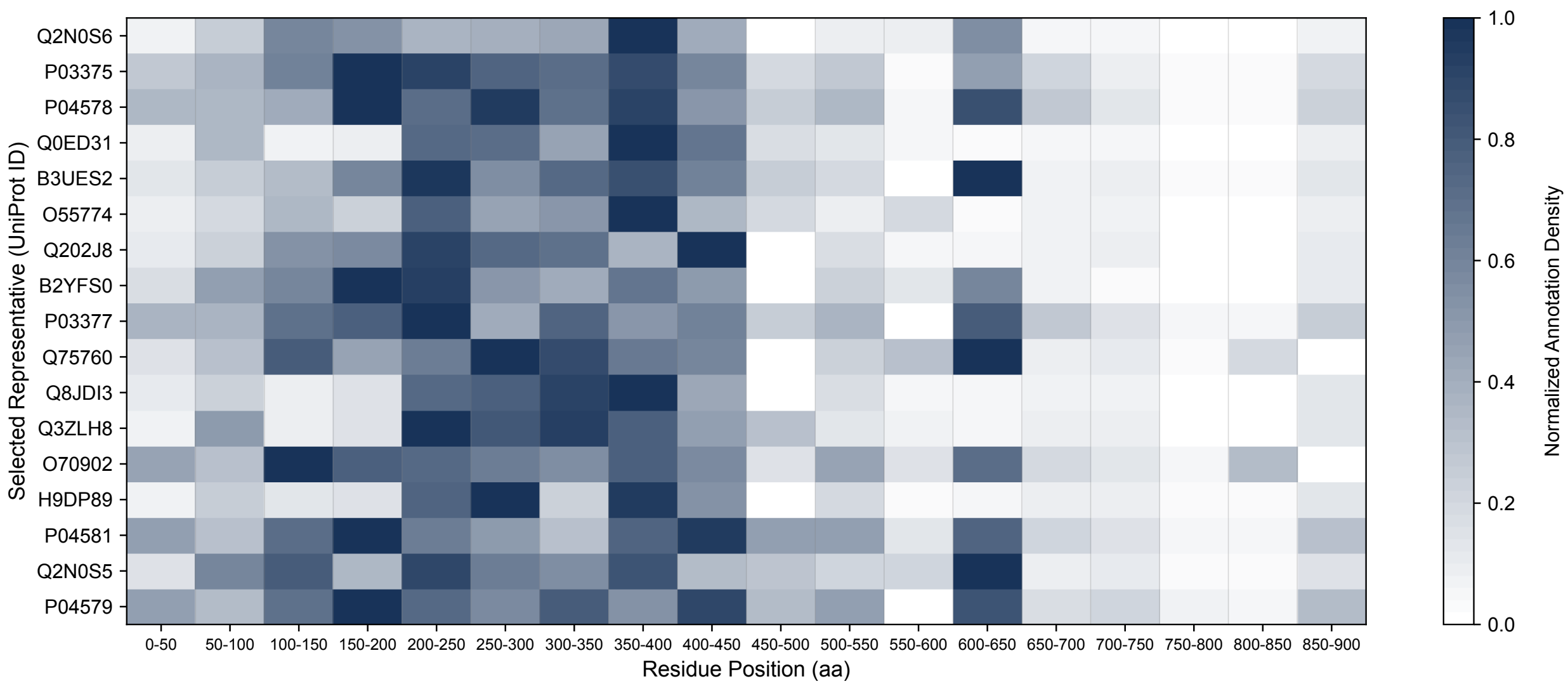

Supplementary Figure 5. Annotation coverage across selected HIV-1 envelope protein representatives. The heatmap shows combined annotation density across all 10 classes for sequence bins (50 residues each) of the 17 representatives retained from 60,348 original entries. Despite a substantial reduction in cluster size, selected representatives maintain both annotation density and coverage.

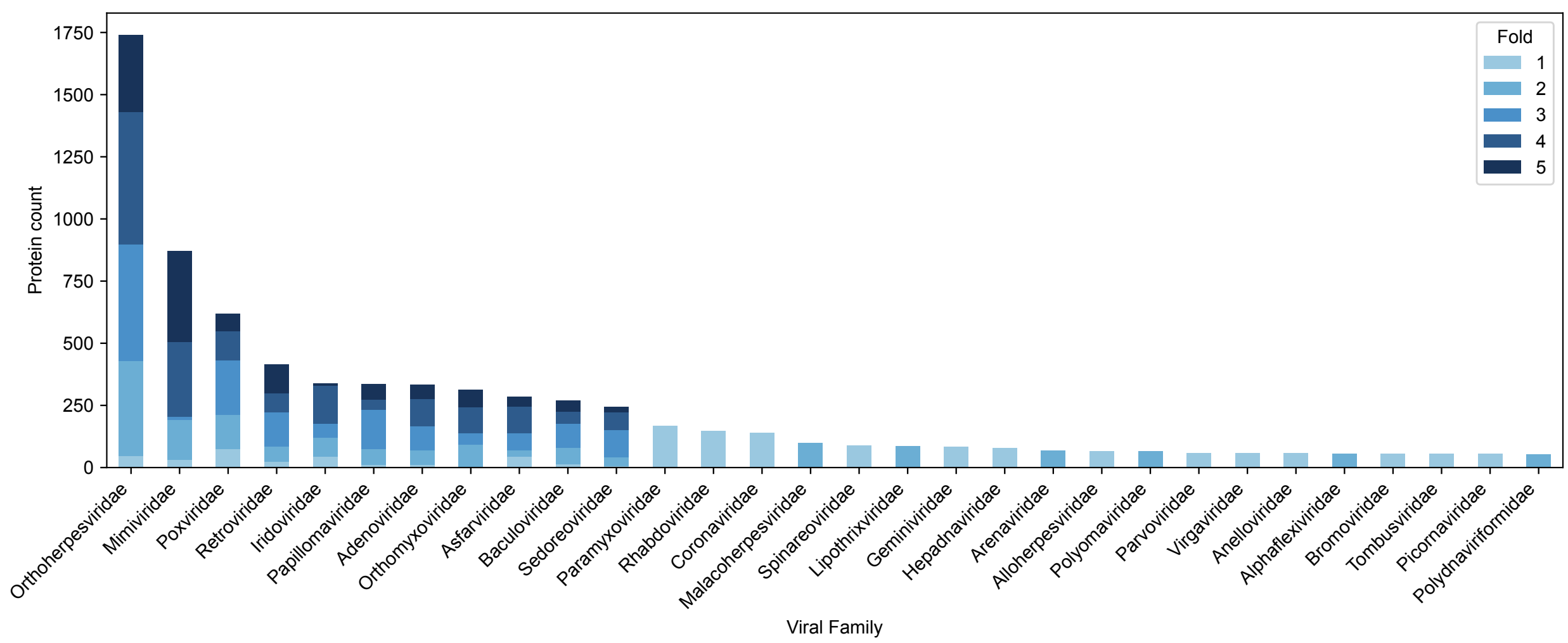

Supplementary Figure 6. Viral family distribution across fold assignments in the final ViralMap dataset, highlighting the top 30 families by protein count, which together account for over 88% of proteins across the 97 unique families in the dataset. Fold assignments aimed to balance annotation class distributions at the residue level while managing taxonomic diversity. Large families (more than 200 proteins) were distributed across multiple folds to prevent any single fold from being dominated by a single taxonomic group. Medium-sized families (20–200 proteins) were each assigned intact to a single fold. Smaller families (fewer than 20 proteins) were assigned at the cluster level; most ended up in a single fold, while those with multiple clusters were distributed across folds as needed to balance residue-level class counts. To prevent homology leakage, clusters were kept intact regardless of family size (no clusters were split across folds).

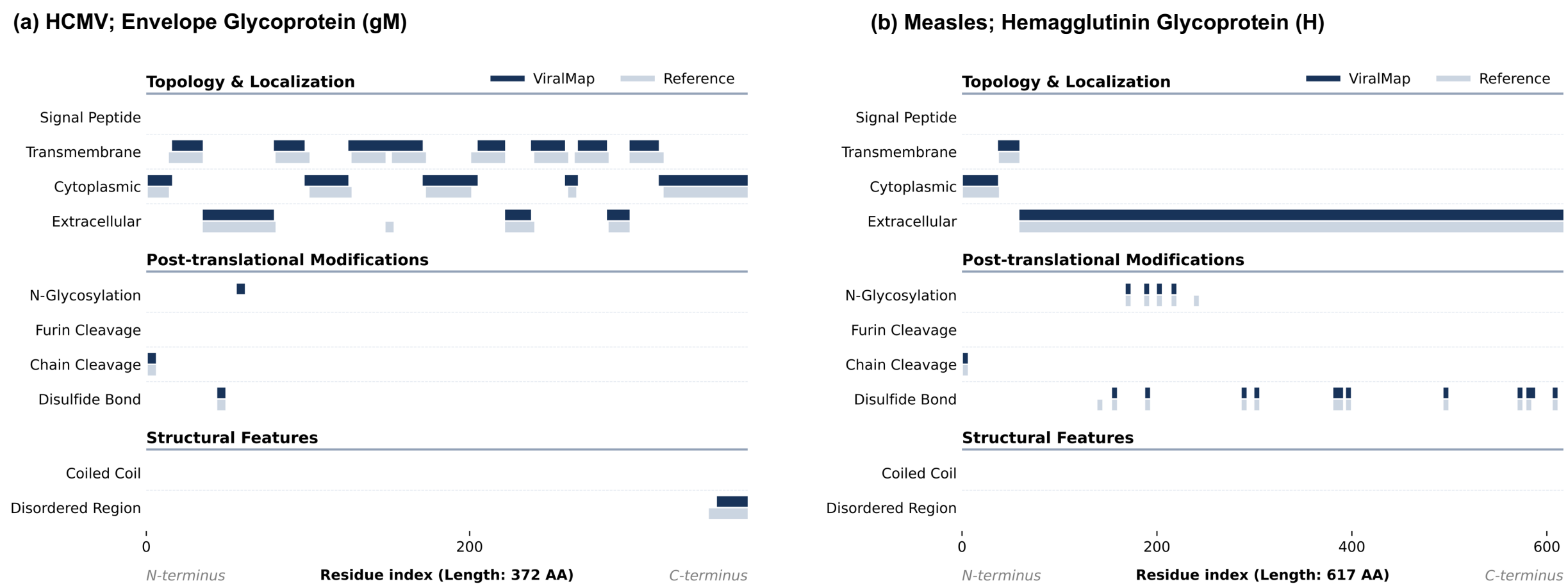

Supplementary Figure 7. ViralMap predictions for additional viral proteins with varying topology. (a) 1D sequence tracks comparing ViralMap predictions against reference annotations for Human cytomegalovirus envelope glycoprotein M, a multi-pass viral envelope protein (UniProt: P16733). (b) 1D sequence tracks comparing ViralMap predictions and reference annotations for Measles virus hemagglutinin glycoprotein, a single-pass Type II membrane glycoprotein (UniProt: P08362). All reference annotations are sourced from UniProt.

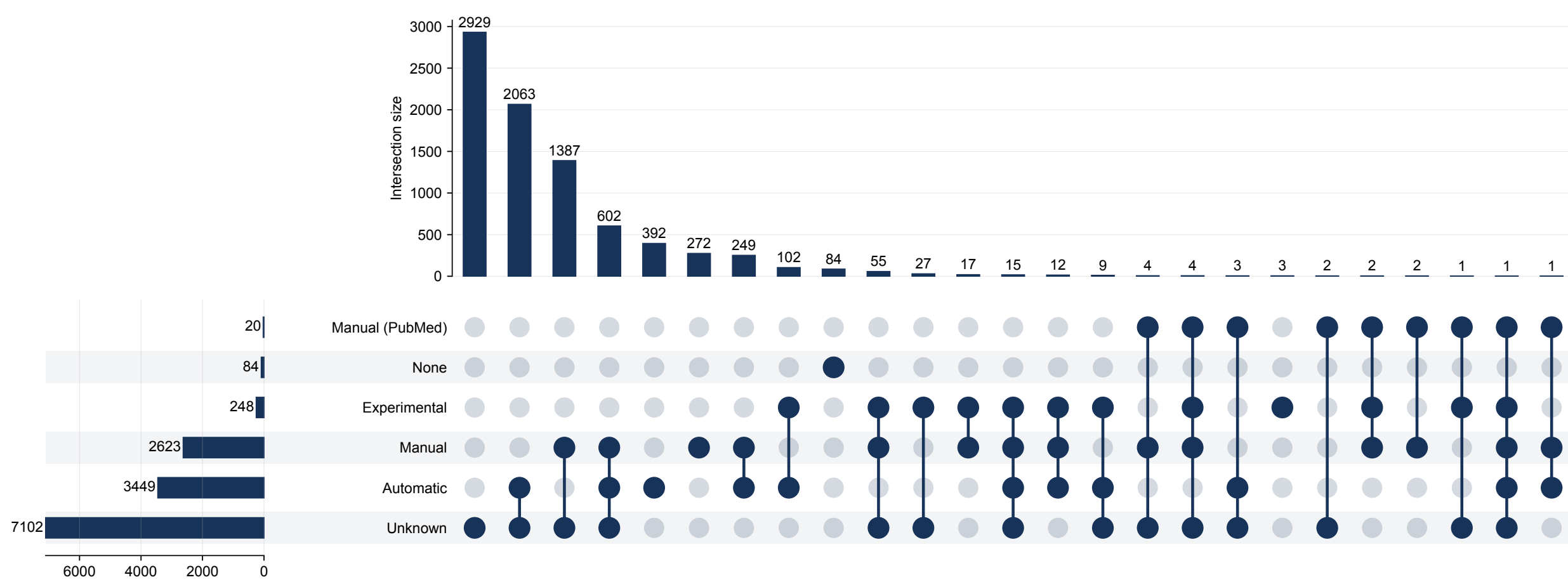

Supplementary Figure 8. Protein-level annotation evidence tier composition across all 8,238 training proteins, sorted by cardinality. The largest intersection (2,929 proteins) contains exclusively Unknown-tier annotations, corresponding largely to proteins such as viral enzymes or nucleoproteins, whose only relevant annotation is chain cleavage and that lack explicit evidence codes in UniProt. These proteins are retained to provide negative examples for proteins with surface-associated annotations like N-glycosylation (Supplementary Table 2).

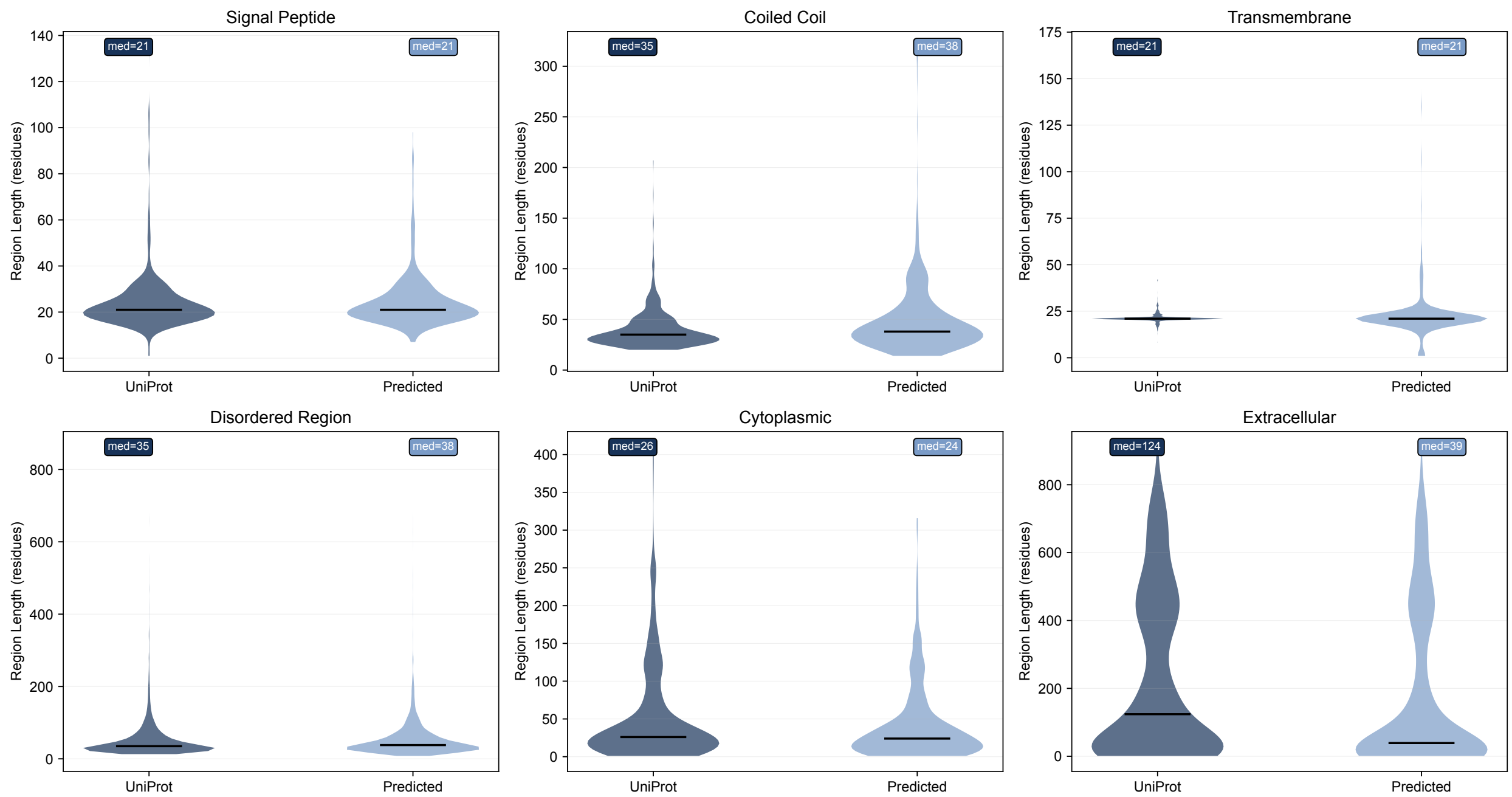

Supplementary Figure 9. Distribution of contiguous region lengths for UniProt annotations and ViralMap predictions across six HMM-decoded annotation classes evaluated on the full test set. Topology classes (Transmembrane, Cytoplasmic, Extracellular) are evaluated only on proteins with at least one topology annotation. Statistics report median length (med, in residues) for UniProt and predicted regions, respectively.

Supplementary Tables

ECO Code	UniProt Description (Abbreviated)	Sources	Type	Score
ECO:0000269	Manually curated information with published experimental evidence	PubMed, Ref.	Experimental	4
ECO:0007744	Combinatorial evidence from experimental and computational sources; manual assertion	PDB	Experimental	4
ECO:0007829	Combinatorial evidence from experimental and computational sources; automatic assertion	PDB	Experimental	4
ECO:0000305	Curator inference based on scientific knowledge or article content	PubMed, None	Manual (Pubmed)	3
ECO:0000250	Propagated from related experimentally characterized protein	None, UniProtKB	Manual	2
ECO:0000255	Sequence model match evidence; curator-verified computational prediction	HAMAP-Rule, None, PROSITE-ProRule	Manual	2
ECO:0000256	Automatic assertion generated by UniProtKB annotation system	HAMAP-Rule, SAM, RuleBase, PIRSR	Automatic	1
-			Unknown	0.5

Supplementary Table 1. Mapping of Evidence and Conclusion Ontology (ECO) codes in UniProt annotations to annotation scores to select cluster representatives and assess dataset quality **(Supplementary Note 1, Supplementary Table 2)**. After the initial filtering, which resulted in 300,406 proteins, 7 unique ECO codes were observed across all annotations in the dataset. By combining the ECO code of a given annotation with the underlying source, we categorized all annotations as belonging to 5 categories: Experimental, Manual (PubMed), Manual, Automatic, or Unknown. All annotations with ECO codes ECO:0007744 and ECO:0007829 originated from experimental PDB entries, which was confirmed by referencing each to the RCSB PDB. ECO codes ECO:0000269, ECO:0007744, and ECO:0007829 were therefore classified as experimental annotations. ECO code ECO:0000305 was categorized as Manual or Manual (PubMed), depending on whether UniProt listed a publication reference with the annotation.

Class	Fold	Experimental	Manual (PubMed)	Manual	Automatic	Unknown	Total
	Fold 1	4 / 87	1 / 19	169 / 3,867	–	6 / 120	180 / 4,093
	Fold 2	5 / 126	1 / 62	145 / 3,774	–	1 / 21	152 / 3,983
SP	Fold 3	8 / 170	–	160 / 3,871	–	2 / 20	170 / 4,061
	Fold 4	1 / 31	–	165 / 3,921	–	4 / 105	170 / 4,057
	Fold 5	3 / 142	–	166 / 3,849	–	6 / 74	175 / 4,065
	**Total**	**21 / 556**	**2 / 81**	**805 / 19,282**		**19 / 340**	**847 / 20,259**
	Fold 1	1 / 66	–	135 / 4,842	3 / 102	–	139 / 5,010
	Fold 2	–	–	118 / 4,433	10 / 489	–	128 / 4,922
CC	Fold 3	8 / 366	–	99 / 4,096	7 / 240	1 / 46	115 / 4,748
	Fold 4	–	–	83 / 3,717	38 / 1,225	–	121 / 4,942
	Fold 5	–	–	112 / 4,551	13 / 447	–	125 / 4,998
	**Total**	**9 / 432**		**547 / 21,639**	**71 / 2,503**	**1 / 46**	**628 / 24,620**
	Fold 1	13 / 294	2 / 42	603 / 12,768	33 / 722	–	651 / 13,826
	Fold 2	1 / 16	–	578 / 12,084	34 / 827	–	613 / 12,927
TM	Fold 3	1 / 26	–	552 / 11,691	93 / 2,034	1 / 29	647 / 13,780
	Fold 4	–	–	577 / 12,253	83 / 2,010	–	660 / 14,263
	Fold 5	–	1 / 18	600 / 12,678	40 / 994	1 / 24	642 / 13,714
	**Total**	**15 / 336**	**3 / 60**	**2,910 / 61,474**	**283 / 6,587**	**2 / 53**	**3,213 / 68,510**
	Fold 1	75 / 75	4 / 4	1,149 / 1,149	2 / 2	5 / 5	1,235 / 1,235
	Fold 2	66 / 66	2 / 2	1,308 / 1,308	153 / 153	–	1,529 / 1,529
NG	Fold 3	92 / 92	–	1,134 / 1,134	8 / 8	–	1,234 / 1,234
	Fold 4	296 / 296	–	1,025 / 1,025	–	–	1,321 / 1,321
	Fold 5	45 / 45	2 / 2	1,359 / 1,359	–	1 / 1	1,407 / 1,407
	**Total**	**574 / 574**	**8 / 8**	**5,975 / 5,975**	**163 / 163**	**6 / 6**	**6,726 / 6,726**
	Fold 1	1 / 23	3 / 89	4 / 92	1,125 / 50,733	–	1,133 / 50,937
	Fold 2	2 / 33	3 / 85	–	1,032 / 51,468	–	1,037 / 51,586
DR	Fold 3	1 / 23	–	8 / 490	874 / 49,770	–	883 / 50,283
	Fold 4	–	–	–	967 / 50,561	–	967 / 50,561
	Fold 5	–	–	–	966 / 46,724	–	966 / 46,724
	**Total**	**4 / 79**	**6 / 174**	**12 / 582**	**4,964 / 249,256**	**–**	**4,986 / 250,091**
	Fold 1	–	–	20 / 40	–	1 / 2	21 / 42
	Fold 2	1 / 2	–	18 / 36	–	1 / 2	20 / 40
FR	Fold 3	2 / 4	–	18 / 36	–	–	20 / 40
	Fold 4	–	–	9 / 18	11 / 22	–	20 / 40
	Fold 5	–	–	14 / 28	6 / 12	–	20 / 40
	**Total**	**3 / 6**		**79 / 158**	**17 / 34**	**2 / 4**	**101 / 202**
	Fold 1	–	–	391 / 391	12 / 12	3,445 / 3,445	3,848 / 3,848
	Fold 2	–	4 / 4	587 / 587	266 / 266	2,982 / 2,982	3,839 / 3,839
CH	Fold 3	2 / 2	–	611 / 611	129 / 129	2,919 / 2,919	3,661 / 3,661
	Fold 4	2 / 2	–	419 / 419	496 / 496	2,762 / 2,762	3,679 / 3,679
	Fold 5		–	397 / 397	57 / 57	2,694 / 2,694	3,148 / 3,148
	**Total**	**4 / 4**	**4 / 4**	**2,405 / 2,405**	**960 / 960**	**14,802 / 14,802**	**18,175 / 18,175**
	Fold 1	214 / 214	1 / 1	1,258 / 1,258	27 / 27	2 / 2	1,502 / 1,502
	Fold 2	393 / 393	3 / 3	1,007 / 1,007	110 / 110	21 / 21	1,534 / 1,534
DB	Fold 3	292 / 292	4 / 4	1,141 / 1,141	14 / 14	13 / 13	1,464 / 1,464
	Fold 4	617 / 617	–	683 / 683	285 / 285	6 / 6	1,591 / 1,591
	Fold 5	513 / 513	2 / 2	944 / 944	115 / 115	19 / 19	1,593 / 1,593
	**Total**	**2,029 / 2,029**	**10 / 10**	**5,033 / 5,033**	**551 / 551**	**61 / 61**	**7,684 / 7,684**
	Fold 1	8 / 467	3 / 181	255 / 15,478	5 / 155	–	271 / 16,281
	Fold 2	3 / 116	5 / 303	295 / 12,509	36 / 1,166	4 / 32	343 / 14,126
CY	Fold 3	1 / 24	1 / 42	286 / 11,583	8 / 953	16 / 1,512	312 / 14,114
	Fold 4	3 / 352	–	178 / 10,715	11 / 1,717	1 / 12	193 / 12,796
	Fold 5	1 / 77	–	257 / 12,724	6 / 948	3 / 591	267 / 14,340
	Total	**16 / 1,036**	**9 / 526**	**1,271 / 63,009**	**66 / 4,939**	**24 / 2,147**	**1,386 / 71,657**
	Fold 1	7 / 189	2 / 79	268 / 73,011	2 / 413	–	279 / 73,692
	Fold 2	1 / 16	5 / 129	270 / 60,552	19 / 662	4 / 576	299 / 61,935
EX	Fold 3	1 / 499	1 / 235	301 / 58,365	8 / 224	14 / 2,050	325 / 61,373
	Fold 4	4 / 249	–	173 / 45,372	1 / 652	2 / 492	180 / 46,765
	Fold 5	1 / 10	–	250 / 62,377	–	3 / 11	254 / 62,398
	**Total**	**14 / 963**	**8 / 443**	**1,262 / 299,677**	**30 / 1,951**	**23 / 3,129**	**1,337 / 306,163**
	Fold 1	323 / 1,415	16 / 415	4,252 / 112,896	1,209 / 52,166	3,459 / 3,574	9,259 / 170,466
	Fold 2	472 / 768	23 / 588	4,326 / 96,290	1,660 / 55,141	3,013 / 3,634	9,494 / 156,421
**Total**	Fold 3	408 / 1,498	6 / 281	4,310 / 93,018	1,141 / 53,372	2,966 / 6,589	8,831 / 154,758
	Fold 4	923 / 1,547		3,312 / 78,123	1,892 / 56,968	2,775 / 3,377	8,902 / 140,015
	Fold 5	563 / 787	5 / 22	4,099 / 98,907	1,203 / 49,297	2,727 / 3,414	8,597 / 152,427
	**Total**	**2,689 / 6,015**	**50 / 1,306**	**20,299 / 479,234**	**7,105 / 266,944**	**14,940 / 20,588**	**45,083 / 774,087**

Supplementary Table 2. Instance and residue counts stratified by evidence source and cross-validation fold for all 10 classes in the final ViralMap dataset: signal peptides (SP), coiled coils (CC), transmembrane domains (TM), N-glycosylation (NG), intrinsically disordered regions (DR), furin cleavage sites (FR), chain cleavage sites (CH), disulfide bond sites (DB), cytoplasmic domains (CY), and extracellular domains (EX). Each entry is formatted as instance count/residue count, where instance count is the number of individual examples of the annotation. For example, the dataset includes 21 signal peptides with experimental evidence.

Tool	Classes	Raw Output	Binarization
DeepTMHMM	SP, TM, CY, EX	Per-residue topology labels: Inside (I), Membrane (M), Outside (O), Signal (S)	Direct mapping: I→CY, M→TM, O→EX, S→SP
DeepCoil	CC	Sharpened per-residue probabilities from peak-finding algorithm; non-peak residues assigned 0, peak residues share maximum	Contiguous regions called positive where peak maximum ≥0.5
AIUPred	DR	Per-residue disorder probabilities	Residues with probability >0.5 called positive; gaps of ≤3 residues between positive regions filled
NetNGlyc	NG	Per-site potential scores for Asn residues in N-glycosylation motifs	Sites with potential >0.5 called positive
ProP	FR	Binary site calls for furin cleavage motifs on the residue preceding cleavage only	Residues flagged as "ProP" are labeled positive (P1, the residue immediately preceding the cleavage bond). UniProt annotates furin cleavage using both flanking residues (P1 and P1'); to match this convention, the residue immediately following each ProP-predicted cleavage was also labeled positive.

Supplementary Table 3. Converting benchmark tool raw outputs into binary predictions for comparison with ViralMap for eight classes: signal peptides (SP), transmembrane domains (TM), cytoplasmic domains (CY), extracellular domains (EX), coiled coils (CC), intrinsically disordered regions (DR), N-glycosylation (NG), and furin cleavage sites (FR).

	Dataset			Performance (residue-level)			
				ViralMap		Benchmark (NetNGlyc)	
**Context**	Total Count	N-Glycosylated?	% of Total	Precision	Recall	Precision	Recall
All Asparagines (Asn)	164,751	6,649	4.04%	**0.651±0.06**	**0.915±0.03**	0.270±0.02	0.829±0.01
N-X-S/T Motifs	25,432	6,622	26.04%	**0.671±0.06**	**0.918±0.03**	0.283±0.02	0.832±0.01
Non-Motif Asn	139,319	27	0.02%	**0.006±0.01**	**0.050±0.10**	–	–

Supplementary Table 4. N-glycosylation prediction performance stratified by asparagine context using F2-optimized thresholds for ViralMap. Dataset columns report counts summed across 5 test folds. Precision and recall are mean ± standard deviation. Bold indicates better performance. NetNGlyc predicts on sequons by default; non-motif performance was not evaluated. Total N-glycosylation site counts are slightly lower than those in Supplementary Table 2 because counts are aggregated across cleaned test folds, where a small subset of proteins was removed to enforce the clustering homology threshold. Bold values indicate the better-performing method for that metric.

	Dataset			Performance (residue-level)			
				ViralMap		Benchmark (ProP)	
**Context**	Motif Count	Cleaved?	% of Total	Precision	Recall	Precision	Recall
R-X-K/R-R↓	2,907	93	3.2%	**0.502±0.11**	0.792±0.36	0.052±0.01	**0.913±0.09**

Supplementary Table 5. Furin cleavage site characterization and prediction (ViralMap F2-optimized thresholds) performance at the canonical motif sequence R-X-K/R-R↓, where X is any amino acid and ↓ is where cleavage occurs. Dataset columns report the overall motif count summed over 5 test folds, and the count/percentage of those motifs that are annotated as cleaved (positive furin cleavage sites) in UniProt. Of the 101 total annotated furin cleavage sites in the dataset **(Supplementary Table 2)**, 93 (92%) fall within this canonical motif. Out of the remaining 8, 1 was removed from the test folds to enforce homology separation **(Methods)**, and the remaining 7 correspond to other motifs and are excluded from the table. Residue-level precision and recall are evaluated over the P1 and P1' residues of canonical motif sites, reported as mean ± standard deviation across test sets. Bold values indicate the better-performing method for that metric.

	Dataset			Performance (residue-level)	
**Context**	Total Count	Disulfide Bond?	% of Total	Precision	Recall
Cysteine	64,852	7,522	11.6%	0.545±0.07	0.869±0.10

Supplementary Table 6. ViralMap disulfide bond prediction performance on cysteine (Cys) residues using F2-optimized thresholds. Dataset counts reflect the total counts across all 5 test folds. Residue-level precision and recall are reported for ViralMap disulfide bond site prediction on cysteines only as mean ± standard deviation across folds. Disulfide bond counts are slightly lower than in **Supplementary Table 2** due to the removal of a small subset of proteins **(Methods)** to enforce the clustering homology threshold.

	Dataset		Performance (residue-level)	
**Context**	Proteins	Cleavage Sites	Precision	Recall
All chain proteins	7,845	17,969	0.896±0.04	0.884±0.02
Single chain proteins	7,003	14,006	0.922±0.02	0.964±0.01
Multi-chain proteins	842	3,963	0.784±0.10	0.593±0.09
Internal sites only	842	2,599	0.651±0.13	0.389±0.10

Supplementary Table 7. Chain cleavage site characterization and prediction performance, stratified by single-chain proteins and multi-chain proteins using F2-optimized thresholds. Single-chain proteins have one pair of boundary positions (start and end of the mature polypeptide), while multi-chain proteins contain additional internal processing sites between mature polypeptide subunits. The dataset columns report the number of proteins and annotated cleavage sites (residue-level), summed across 5 test folds. Precision and recall are reported as mean ± standard deviation across 5 test folds. No benchmark tool was available for chain cleavage prediction. Only proteins with chain annotations in UniProt are included

in calculating performance. Cleavage site counts are slightly lower than in **Supplementary Table 2** due to the removal of a small subset of proteins **(Methods)** to enforce the clustering homology threshold.

Feature	ViralMap PR-AUC	ViralMap Precision	ViralMap Recall	Benchmark Tool	Benchmark Precision	Benchmark Recall
Post-translational Modifications						
N-Glycosylation	0.763±0.06	**0.701±0.06**	**0.844±0.07**	(a)	0.270±0.02	0.827±0.01
Furin Cleavage	0.565±0.16	**0.506±0.13**	0.690±0.31	(b)	0.031±0.003	**0.880±0.07**
Chain Cleavage	0.895±0.02	**0.918±0.01**	**0.859±0.03**	–	–	–
Disulfide Bond	0.754±0.03	**0.644±0.04**	**0.733±0.11**	–	–	–

Supplementary Table 8. Baseline classification performance of ViralMap and benchmark tools: **(a)** NetNGlyc **(b)** ProP. This table presents ViralMap's performance using a standard 0.5 probability threshold for all post-translational modification classes (N-glycosylation, furin cleavage, chain cleavage, and disulfide bonds) rather than the F2-optimized thresholds reported in **Table 1**. All metrics are reported as mean ± standard deviation across five test folds. A bold value designates the higher precision or recall metric between ViralMap and the benchmark tool for a given annotation class.

Annotation Class	All Test Data Precision	All Test Data Recall	Family Holdout & Identity ≤30% Residues	Family Holdout & Identity ≤30% Precision	Family Holdout & Identity ≤30% Recall
Topology & Localization					
Signal Peptide	0.675	0.843	*3,400*	0.613	0.829
Transmembrane	0.881	0.868	*16,840*	0.840	0.808
Cytoplasmic	0.929	0.698	*21,471*	0.926	0.562
Extracellular	0.957	0.937	*87,765*	0.946	0.936
Post-translational Modifications					
N-Glycosylation	0.644	0.915	*1,480*	0.681	0.918
Furin Cleavage	0.391	0.780	*56*	0.516	0.571
Chain Cleavage	0.857	0.884	*4,667*	0.840	0.871
Disulfide Bond	0.519	0.871	*1,686*	0.421	0.795
Structural Features					
Coiled Coil	0.485	0.539	*6,272*	0.386	0.516
Disordered Region	0.832	0.893	*67,649*	0.850	0.869

Supplementary Table 9. Micro-averaged residue-level precision and recall for ViralMap on the full test set and on 2,118 family-holdout proteins whose maximum sequence identity to any training protein is ≤30% at any alignment coverage. Post-translational modifications use F2-optimized thresholds. Topology and localization and structural features use HMM decoding. Residues indicate the number of positively labeled residues in the holdout subset. The full test set metrics are recomputed using the same pooled micro-averaging approach for direct comparability with the holdout subset.

	All Test Data (excl. Unknown/None)		
**Annotation Class**	Residues	Precision	Recall
Topology & Localization			
Signal Peptide	*20,130*	0.717	0.843
Transmembrane	*67,781*	0.881	0.868
Cytoplasmic	*70,881*	0.929	0.698
Extracellular	*304,128*	0.957	0.937
Post-translational Modifications			
N-Glycosylation	*6,663*	0.670	0.915
Furin Cleavage	*200*	0.391	0.780
Chain Cleavage	*12,002*	0.817	0.842
Disulfide Bond	*7,518*	0.537	0.871
Structural Features			
Coiled Coil	*24,336*	0.544	0.539
Disordered Region	*248,416*	0.849	0.893

Supplementary Table 10. Micro-averaged residue-level precision and recall for ViralMap evaluated on 5,156 test proteins whose highest annotation evidence tier is Automatic or above, excluding proteins with only Unknown-tier or no annotations. Micro-averaged full-test-set metrics are reported in Supplementary Table 9 for comparison. Post-translational modifications use F2-optimized thresholds. Topology and localization and structural features use HMM decoding. Residues indicate the number of positively labeled residues in the holdout subset.

Supplementary Notes

Supplementary Note 1: Annotation vector representative selection

The filtered dataset of 300,406 proteins contained substantial redundancy with certain clusters dominating (e.g., >60,000 entries for HIV-1 envelope protein). Retaining all entries would introduce excessive label noise into model training, while random down-sampling risked removing well-annotated proteins or sparsely annotated proteins containing rare annotation classes. We therefore implemented Algorithm 1 below to select representatives from each cluster, maximizing annotation quality and coverage while minimizing redundancy. To compare quality and coverage across entries, all proteins were represented as fixed-length annotation vectors of 210 dimensions (10 annotation classes × 21 positional bins). This length was determined using the longest protein in the dataset (1,024 residues after the length filter). Using a fixed vector length for all proteins did not have a significant impact on selection since clusters were largely homogenous in length **(Supplementary Fig. 3)**. Each protein's annotation vector was generated by converting all annotations into scores based on their Evidence and Conclusion Ontology (ECO) codes provided in UniProt, then assigning the scores to bins corresponding to the positions of the annotations. Scores were determined based on a five-point scale: experimentally validated annotations received score 4; manual annotations with PubMed references received score 3; manual annotations without PubMed references received score 2; automatic annotations received score 1; and annotations without ECO codes received 0.5. Mapping ECO codes to these annotation scores or tiers is described in **Supplementary Table 1**. Once populated, these annotation vectors had a dual benefit: their magnitudes (L2 norms) captured overall annotation quality, while their directions indicated whether a given protein contained an annotation at a position missing in other representatives. Both norm and direction were used in Algorithm 1 to select representative proteins. Candidate proteins were scored by an 'information gain' metric combining the annotation vector norm and cosine similarity with already selected representatives. The cosine similarity (direction) was used to maximize coverage, since proteins that contributed new information by annotating positions missing in other representatives would, in principle, have lower similarity. This iterative selection continued within each cluster until a hard cap was reached or no remaining protein had annotations that would contribute new information (as measured by a minimum information gain threshold of 1.0, or the equivalent of a single automatic annotation). This reduced the dataset from 300,406 to 8,238 proteins while primarily removing proteins contributing automatic annotations (**Supplementary Fig. 4**). The HIV-1 envelope glycoprotein cluster, for instance, was reduced from 60,348 entries to 17 representatives **(Supplementary Fig. 5)**

Algorithm 1

1: **Input:** Proteins with residue-level annotations across $C = 10$ classes (SP, CC, TM, NG, DR, FR, CH, DB, CY, EX), each with an associated evidence code, and a sequence-identity cluster identifier for each protein.

2: **Construct annotation vectors:**
- ▷ Divide each protein into $B = 21$ bins ($\lceil \frac{1024}{50} \rceil$) of 50 residues each
- ▷ For each protein p, initialize annotation vector $\mathbf{v}_p \in \mathbb{R}^{210}$ (10 classes × 21 bins)
- ▷ For each annotation, increment the corresponding bin(s) by evidence weight w: experimental = 4.0, manual/PubMed = 3.0, manual = 2.0, automatic = 1.0, unknown = 0.5
- ▷ Compute quality score $q_p = \|\mathbf{v}_p\|_2$

3: **Define cluster representative cap:**
- ▷ For cluster of size n, set $k_{\max} = \min\big(100, \lceil \log_2(n) \rceil + 1\big)$

4: **Greedy selection within each cluster:**
- ▷ Initialize selected set $S \leftarrow \emptyset$
- ▷ While $|S| < k_{\max}$:
  - – For each candidate p not in S, compute novelty $\text{nov}_p = \max_{s \in S} \cos(\mathbf{v}_p, \mathbf{v}_s)$ (0 if $S = \emptyset$)
  - – Compute gain $g_p = \alpha \cdot q_p + \beta \cdot (1 - \text{nov}_p)$ with $\alpha = 1.0$, $\beta = 3.0$
  - – Select $p^\star = \arg\max_p g_p$
  - – If $g_{p^\star} < \tau = 1.0$, terminate early
  - – Otherwise, add $p^\star$ to S

5: **Output:** Selected representatives from each cluster, prioritizing proteins with high-quality and non-redundant annotations.
